# Rhabdomyolysis Associated With “Pruno” Prison-Made Alcohol Intake in Corrections Facility: A Case Report

**DOI:** 10.7759/cureus.30933

**Published:** 2022-10-31

**Authors:** Mary H Soares, Guillermo Izquierdo-Pretel, Lauren A Ramesar

**Affiliations:** 1 Medicine, Florida International University, Herbert Wertheim College of Medicine, Miami, USA; 2 Internal Medicine, Florida International University, Herbert Wertheim College of Medicine, Miami, USA

**Keywords:** alcohol withdraw in prison, hyperactive delirium, “pruno”, prison-made alcohol, rhabdomyolysis

## Abstract

In this study, a case of severe rhabdomyolysis in a 28-year-old incarcerated patient is presented. The patient initially presented with emesis, abdominal pain, and myalgias to the clinic at the corrections facility and was treated with antiemetics and analgesics. The onset of symptoms occurred approximately 24 h after ingestion of an illicit substance produced by inmates. Despite intervention, the patient was eventually transferred to the hospital on the third day after symptom onset for further evaluation and treatment. The manuscript presents the events that preceded the diagnosis of rhabdomyolysis, as well as symptoms of hyperactive delirium that developed during the patient's hospital course, leading to the high suspicion of illicit substance intoxication as a contributing inciting factor of rhabdomyolysis. This study aimed to bring awareness to the medical community about substance use in the correction system and its consequences.

## Introduction

Rhabdomyolysis is caused by the lysis of striated muscle leading to the release of intracellular content into the bloodstream from necrotic muscle tissue. It can occur in both sexes and all age groups via traumatic injuries, such as strenuous activities and seizures, or non-traumatic injuries to muscles, such as alcohol, infections, and electrolyte imbalance [1]. Depending on the extent of the damage-causing muscle cell edema, rhabdomyolysis has a broad range of manifestations from an asymptomatic rise in creatine kinase to medical emergencies where patients present with diffuse myalgias, weakness, and sometimes dark urine with brown casts [2].

Rhabdomyolysis can lead to severe consequences including acute renal failure and can become life-threatening [3,4]. Rhabdomyolysis is a relatively common occurrence, with a national incidence of 26,000 annual cases, and is often attributed to vigorous exercise when present in incarcerated individuals [5]. The consumption of an alcoholic substance produced from fermented fruits and bread, water, sugar, potato, and other miscellaneous ingredients by inmates often called “pruno,” “hooch,” or “prison wine” has been documented in the literature [6]. Ingestion of this substance has been associated with several botulism outbreaks in prison, including maximum security facilities [7-10]. In this case report, a patient where consumption of prison-produced alcohol was believed to be associated with rhabdomyolysis is discussed. We hope to contribute to the limited existing literature on this matter and raise awareness of other inciting factors that may lead to rhabdomyolysis and its severe and potentially life-threatening consequences.

## Case presentation

Three days before admission to the hospital

A 28-year-old inmate with a past medical history of migraines and social history significant for daily strength training was found vomiting by a dorm officer and was then taken to the corrections health services, where he received an antiemetic before returning to his cell. The patient was accustomed to a daily exercise routine since adolescence.

Two days before admission to the hospital

The patient was seen once again by a physician at corrections health services with complaints of generalized body aches and cramping. Physician documentation reported that the patient was visibly diaphoretic and complaining of abdominal pain. The patient stated that he had soup that was made in the unit and began having abdominal pain and cramping, which led to vomiting. He also reported having his usual workout routine the day before. His vitals were as follows: heart rate (HR) of 82 beats per minute (bpm), respiratory rate (RR) 20 breaths per minute, blood pressure (BP) of 147/91 mmHg, and he was in moderate distress. Abdominal examination was significant for diffuse tenderness to palpation with the abdomen being soft, non-distended, with normoactive bowel sounds. He received ondansetron 4 mg sublingually three times daily (TID) every 6 h as needed and was encouraged to drink plenty of fluids.

In the afternoon, the patient had complaints of intermittent muscle spasms and returned to the clinic. He received Toradol 30 mg intramuscularly (IM) with mild relief. He was also experiencing upper epigastric pain radiating to the back, and muscle spasms on thighs and calves bilaterally. No nausea or vomiting. Vitals were significant for elevated BP 161/87 mmHg. His abdominal examination findings remained the same with diffuse tenderness to palpation, abdomen soft, non-distended, with normoactive bowel sounds. The patient remained at the clinic for observation. 

In the evening, the patient reported that he was experiencing worsening lower extremity pain and headache and was diaphoretic. He reported having his last bowel movement the day before. Headache was not relieved with Tylenol. Vital signs on physical examination revealed heart rate of 102 bpm, respiratory rate of 20 breaths per minute, and blood pressure of 129/93 mmHg.

One day before admission to the hospital

The patient continued to experience muscle spasms in his arms and legs, abdominal pain, and one episode of non-bloody emesis. He denied fever, shortness of breath, chest pain, dizziness, or palpitations. The physician at the Corrections Health Services ordered labs including creatine phosphokinase (CPK), complete blood count (CBC) with differential, thyroid-stimulating hormone (TSH), and complete metabolic panel (CMP). He was given cyclobenzaprine 10 mg orally twice a day (BID) and Gatorade for hydration.

Day of admission

The patient continued to experience muscle spasms and significant abdominal pain without resolution from cyclobenzaprine and oral hydration. The patient's lab results were significant for elevated creatine phosphokinase (CPK) 26,638 units/L, blood urea nitrogen (BUN) 22 mg, and creatinine 1.8 mg/dL, which prompted the administration of a normal saline (NS) intravenous (IV) bolus. This intervention led to no improvement in symptoms, which prompted the patient to be transferred to the emergency department (ED) for further evaluation and high acuity care for rhabdomyolysis with acute kidney injury (Table 1).

**Table 1:** Laboratory results at three timepoints during hospital course. *Laboratory tests were not repeated during hospital course. BUN: blood urea nitrogen; TSH: thyroid-stimulating hormone; CPK: creatine phosphokinase

Laboratory	Admission time results	Agitation time results	Discharge time results	Reference values
White blood cells count	11.5	6.0	5.6	4.0-10.5 x 10^3^/mcL
Red blood cells count	5.74	5.51	5.14	4.2-5.6 x 10^6^/mcL
Hemoglobin	13.0	13.0	12.2	13.3-16.3 g/dL
Hematocrit	43.2	41.1	38.2	39.0-47.1%
Platelet count	207	208	193	140-400 x10^3^/mcL
Whole blood glucose	90	90	74	74-106 mg/dL
Whole blood sodium	134	137	136	137-145 mmol/L
Whole blood potassium	4.3	4.0	4.1	3.6-5.0 mmol/L
BUN level	20	18	14	9-20 mg/dL
Carbon dioxide (CO_2_)	26	28	24	22-30 mmol/L
Creatinine level	1.8	1.2	1.1	0.52-1.04 mg/dL
Calcium level	8.7	9.3	8.8	8.4-10.2 mg/dL
Total protein	8.9	7.1	6.9	6.3-8.2 g/dL
Albumin level	5.2	4.0	4.0	3.9-5.0 g/dL
Aspartate aminotransferase (AST)	517	181	67	15-46 unit/L
Alanine transaminase (ALT)	108	94	65	21-72 unit/L
TSH*	1.72	-	-	0.27-4.22 mIU/mL
C-reactive protein*	<0.5	-	-	0.0-0.9 mg/dL
CPK	26,758	22,467	871	57-374 units/L

At the ED, he received aggressive hydration with 4 L of NS IV, and an ultrasound of the abdomen was ordered. The hospitalist service was consulted for admission and further management. Upon inquiry, the patient denied alcohol use, smoking, or other substance use such as steroids. Urine drug screen was negative. Abdominal ultrasound showed echogenic liver likely reflecting steatosis but was otherwise unremarkable.

Day two of admission

The hospitalist service attending was informed that the patient was agitated and restless overnight with no sleep. He was given haloperidol 5 mg IV at 9:17 am. Approximately 1 h later, the patient became increasingly agitated and diaphoretic. A rapid response team was called, and the patient was found to be restless and agitated. Correctional officers present at the bedside secured the patient by handcuffs on all four extremities. The patient removed his peripheral IV lines twice. He was disoriented four times, unable to respond to questions, and appeared to be hallucinating. Corrections documentation did not show any prior history of psychiatric illness, medications, or substance use disorder. We were unable to rule out substance use in corrections, despite negative toxicology screen. Ethanol level four days after onset of symptoms was <10 mg/dL (normal range: 0-9 mg/dL). No presence of other types of alcohol including methanol or isopropanol. He was given lorazepam 2 mg IV at 10:04 am with no improvement. The patient continued to be restless in his handcuffs, which increased the risk of injury and worsening rhabdomyolysis. The patient calmed down after an additional 2 mg of lorazepam was given at 12:22 pm. Peripheral IV line was reinserted, and 1 L normal saline IV bolus was initiated. His vital signs were as follows: temperature 37.1°C, HR 78 bpm, RR 20 breaths per minute, BP 124/72 mmHg, and SpO_2_ 99% on room air.

Psychiatry was consulted and found the patient to be markedly restless, appearing to be picking up imaginary objects from the air and placing it in his mouth. He was also found to be talking to himself and responding to internal stimuli. Psychiatry recommended the administration of valproic acid 500 mg for agitated delirium, Zyprexa 5 mg at bedtime for agitated delirium and disorganized thoughts, and Zyprexa IM for severe agitation. The patient was then transferred to the video monitoring floor. Due to marked restlessness and agitation, the patient was started on IV medication including lorazepam, haloperidol, and valproic acid. 

Day three of admission

The patient was seen at the bedside and was found to be calm and able to interact with the attending physician. He reported one episode of emesis in the morning but otherwise tolerated liquids and Ensure Enlive. When inquired about events leading up to the onset of symptoms, the patient stated that he had completed his regular workout routine and acknowledged poor hydration. When asked about substance use, the patient reported that he had consumed a substance produced by inmates often called “pruno,” “hooch,” or “prison wine” made from fermented fruits and bread. He reported that he had consumed this substance for three consecutive days prior to the onset of symptoms. He denied steroid or other substance use.

Days four to seven of admission

**Figure 1:**
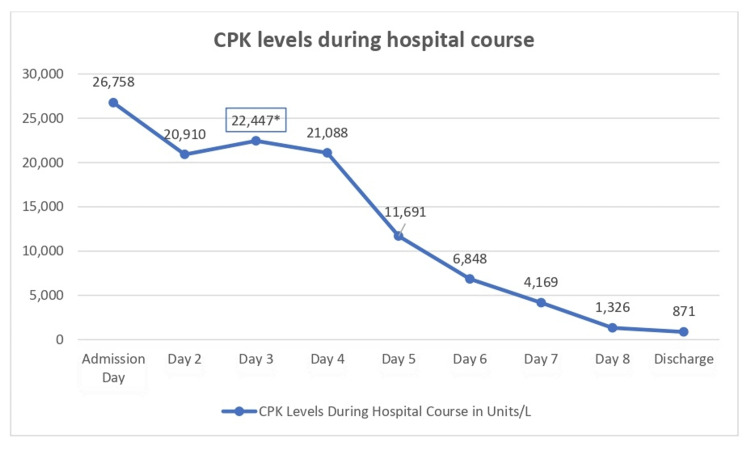
CPK level trend during hospital course. *CPK level after period of agitation. CPK: creatine phosphokinase

The patient continued to receive aggressive IV and oral hydration for additional four days. The patient regained baseline mental status with no complications. Repeated laboratory tests prior to discharge showed BUN 13 mg/dL and creatinine level 1.10 mg/dL within normal limits, and improved liver function tests (LFTs) with AST 67 unit/L and ALT 65 unit/L. CPK level at the time of discharge was 871 units/L (see Figure 1 for CPK level trend). His vital signs were as follows: temperature 37°C, HR 86 bpm, BP 136/74 mmHg, RR 20 breaths per minute, and SpO_2_ 98% on room air. The patient was then discharged to the corrections facility.

## Discussion

The patient in this case presentation was diagnosed with rhabdomyolysis based on his progressive abdominal pain, muscle spasms, and significantly elevated CPK present in laboratory results. His symptomatology developed after consuming prison-produced wine for three consecutive days prior to the onset of symptoms, which the patient established as the only potential inciting factor that differed from his normal routine.

Rhabdomyolysis is characterized by damage to muscle cells where its necrotic content enters the bloodstream and often causes kidney injury [1,2]. It can have serious consequences including acute kidney failure and death with several fatal cases reported in the literature [3,4]. Rhabdomyolysis cases in the corrections system are often attributed to overexertion during exercise [5]. However, consumption of illicitly produced alcoholic substances should also be investigated as the possible causative agent of rhabdomyolysis in this patient population.

The case presented here illustrates a muscular patient accustomed to a daily exercise routine since adolescence with no prior history of rhabdomyolysis. This makes exercise less likely to be the sole inciting factor. Other causes of rhabdomyolysis include prescription medications such as lipid-lowering agents, antimicrobials, psychiatric medications, and antihistamines, among others, as well as illicit drugs including cocaine, amphetamines and methamphetamines, heroin, and hallucinogens [1]. These were excluded as patient history and medical records showed that he was not taking any prescription medications and his toxicology screening was also negative except for ethanol <10 mg/dL four days after the onset of symptoms.

Although the mechanism is not fully understood, alcohol-induced rhabdomyolysis may occur with both short- and long-term alcohol intoxication. According to existing literature, it is believed that the toxic effect of alcohol may occur via the disruption of ATP pump leading to the induction of cytochrome P450 and changes to the sarcoplasmic reticulum leading to the breakdown of muscle membrane [11].

The ingestion of illicit prison-brewed alcohol has been documented as the inciting factor in several botulism outbreaks in the corrections system [6-10]. In this case, the patient reported drinking this substance for three consecutive days prior to symptom onset, which raises the likelihood of alcohol-induced rhabdomyolysis. One of the challenges faced with this substance is the fact that its exact composition is often unknown. Ingredients may include canned fruits such as peaches and pears, grapefruit and oranges, water, juice mix, and one- to two-week-old baked potatoes, which are believed to help speed the fermentation process [9]. However, given the limitation in availability of these ingredients, other substitutions are often made increasing the risk of toxicity depending on composition. Unfortunately, there is no specific confirmatory testing. However, we are unable to rule out intoxication or withdrawal from “pruno” as the cause of the hyperactive delirium experienced by the patient in this case.

Additionally, in this case, when the patient developed symptoms of withdrawal, it interfered with the management of rhabdomyolysis, as the patient was severely agitated and repeatedly removed the intravenous line which is vital to adequate treatment.

Illicit production and consumption of “pruno” in prison is common knowledge among both inmates and correction officers. In this case, with the condition of anonymity, an officer present with the patient in the hospital corroborated that the consumption of “pruno” is ubiquitous. Despite alcohol consumption not being expected in this environment, alcohol intoxication should be suspected in all patients with rhabdomyolysis presenting from corrections.

## Conclusions

Rhabdomyolysis is a condition that can lead to serious consequences including death, and it is a common occurrence in corrections. “pruno” ingestion by inmates is ubiquitous and can lead to severe cases of rhabdomyolysis, as well as hyperactive delirium. Our hope is to bring awareness to the medical community about this substance and its impact to health. Knowledge about the use of “pruno” in this patient population is paramount to proper management and prevention of serious outcomes that can be fatal.
